# Reasons for exercise inventory: A validation of the Dutch version in a community sample and a sample of women with an eating disorder

**DOI:** 10.1177/13591053251375314

**Published:** 2025-10-03

**Authors:** Aurélie Nieuwenhuijse, Mia Scheffers, Marlies Rekkers, Jooske T. van Busschbach, Annemarie A. van Elburg

**Affiliations:** 1 Utrecht University, the Netherlands; 2 Windesheim University of Applied Sciences, Zwolle, the Netherlands; 3 Emergis Network EEN, Goes, the Netherlands; 4 University of Groningen, University Medical Center Groningen, Rob Giel Onderzoekcentrum, the Netherlands

**Keywords:** eating disorders, compulsive exercise, reasons for exercise, psychometric evaluation, body satisfaction

## Abstract

The reasons for exercise inventory (REI) is a self-report instrument to assess why people exercise. It is important to assess the reasons for exercise in people with an eating disorder (ED), who regularly suffer from compulsive exercise. In this study the psychometric properties of the Dutch version of the REI and differences in scores are evaluated in an ED and a community sample consisting of women. Factor analysis revealed three factors: health, appearance/weight and mood. Reliability (internal consistency, test-retest) was good. The ED sample scored significantly lower on the Health subscale and significantly higher on the appereance/weight and mood subscales than the community sample exercising for appearance/weight showed a moderately positive correlation with EDE-Q scores in both samples. The Dutch version of the REI showed promising psychometric properties that support its value for clinical and research purposes. Using three subscales may enhance research and assessment of the reasons for exercise.

## Background

Compulsive exercise (CE) is frequently found in patients with eating disorders (EDs). It manifests across different ED diagnoses as a rigid and emotionally driven urge to engage in physical activity, primarily aimed at weight control or affect regulation (Cambel et al., 2025). While CE is most commonly reported in individuals with anorexia nervosa (AN), it is also present in those diagnosed with bulimia nervosa (BN), other specified food and eating disorder (OSFED) and binge eating disorder (BED). Depending on the assessment method, prevalence rates of CE vary and can be as high as 81% in AN and 66% in BN (Dittmer et al., 2018). According to Monell et al. (2018) prevalence rates in women with OSFED are 55% and BED 13%. Although regular physical activity usually improves mental and physical health, CE is linked with worse physical and mental health outcomes, higher severity of ED symptomatology (Dittmer et al., 2018) and higher risk of relapse (Carter et al., 2004). Also, CE is found to be associated with ED features such as drive for thinness, body dissatisfaction (Solenberger, 2001), and weight and shape concerns (Dalle Grave et al., 2008; Meyer et al., 2011). Especially people with an ED diagnosis do not seem to be motivated by general health goals but prioritize changes in body shape and weight (Ryan and Deci, 2017). According to the cognitive and behavioural maintenance model of compulsive exercise of Meyer et al. (2011), patients with EDs not only exercise to control weight and shape concerns, but also use exercise to improve mood and to prevent or regulate negative affect. All in all, when treating eating-disordered patients, it is important to investigate their reasons for exercise. For ED patients with compulsive exercise behaviour or unhealthy exercise reasons, insight into their reasons for exercise can be crucial in helping them to achieve healthier exercise behaviour.

Reasons for exercise can be assessed using a specific self-report questionnaire. The reasons for exercise inventory (REI) is a widely used self-report questionnaire to measure exercise motivations. The original English version of the REI, developed by Silberstein et al. (1988), consisted of 24 items divided into 7 factors and showed adequate internal consistency and validity. Over the years, other studies reported different subscale structures. The psychometric evaluation by Cash et al. (1994), revealed four factors: appearance/weight management, fitness/health management, stress/mood management and socializing in a 25-item version of the REI. Another study by Strelan et al. (2003) reduced the 7 factors of the original 24 items version to three distinct factors: appearance, health and fitness, and enjoyment/mood improvement reasons for exercise. These subscales were also used by Lustyk et al. (2004), O’Hara et al. (2014) and Prichard and Tiggemann (2005, 2008). Based on these studies, the 24-item version with a three-subscale structure now seems to be the best version to apply in research in non-clinical groups. To date we have not found any studies that have psychometrically evaluated the original subscales of the REI in a sample of ED. It has been used in both descriptive and intervention studies in this field (Bratland-Sanda et al., 2010; Harris et al., 2020; Young et al., 2017).

In clinical practice the REI can be used to gain insight into a patient’s reasons for exercise and physical activity behaviour. In addition, the instrument has the potential to measure changes in reasons to exercise as an effect of therapeutic interventions targeting CE. However, since up until now no Dutch version of the REI is available. In the Netherlands and Belgian there is a need for a reliable and valid Dutch version of the REI to be able to evaluate reasons for exercise in ED patients. Currently, there are no validated self report questionnaires available in Dutch that assess compulsive exercise in adults.

In this study, the central aim is to psychometrically evaluate the Dutch version of the REI in a clinical sample consisting of women with ED and a community sample of women. In addition, associations between reasons for exercise, ED symptoms and body satisfaction will be explored in both samples. As stated above, previous studies suggest that severity of ED is especially associated with exercising for weight and shape or mood improvement and a negative evaluation of one’s body (Bratland-Sanda et al., 2010; Meyer et al., 2011). Furthermore, we will evaluate differences in REI scores between the group of clinical ED patients and the community sample.

## Methods

### Procedure

The study protocol was approved by the local ethics committee of the Faculty of Social and Behavioural Sciences of Utrecht University (number 20-383). The Medical Ethics Review Committee of Utrecht University waived the requirement of ethical approvement (Reference number WAG/mb/20/022653). Participation was voluntary and data were analyzed anonymously.

Two independent samples were used in this study: a sample of ED patients in treatment in specialized ED treatment centre and a community sample. The participants in the ED sample were approached between February and November 2021 through posters and flyers featuring the title of the study, ‘Exercise behaviour and body image’, a short text about the study and a QR code leading to the self-report questionnaires, information about the objective of the study and informed consent in a secure online system (Qualtrics.com). The posters and flyers were displayed in the waiting rooms of 13 specialized ED treatment centres across the Netherlands. The community sample was recruited between November 2019 and May 2020 by students of Windesheim University of Applied Sciences, School of Human Movement and Sports Windesheim, using a snowball sampling method. After participants (*n* = 982) provided digital informed consent, they were asked to fill out the questionnaires using an email link or through social media, which led to a secure online system using the web-based programme and server Formdesk.

### Participants

For the ED sample inclusion requirements were a minimum age of 18 years and current treatment in a specialized ED treatment centre. Two hundred and forty-one ED patients gave consent and started the questionnaire of which 175 were filled out completely and therefore suitable for analysis. The final sample consisted of 166 women with ED, 8 men and 1 patient with unidentified gender. For analyses and equitable comparisons, men and gender-neutral participants were excluded from the ED sample resulting in a sample of 166 women with ED, aged between 18 and 67 (*M*_age_ 29.40, SD 10.36). Of these participants, 103 followed an outpatient ED treatment and 63 an inpatient ED treatment. The distribution of self-reported diagnoses according to DSM-5 criteria was as follows: AN 60.2% (*n* = 100); BN 10.2% (*n* = 17), binge eating disorder (BED) 15.1% (*n* = 25), otherwise specified feeding and eating disorder (OSFED) 13.9% (*n* = 23) and avoidance restrictive food intake disorder (ARFID) 0.6% (*n* = 1). The self-reported body mass index (BMI) was *M*_BMI_ 22.45 (SD 7.90, range 12.59–48.65).

During the data collection in the community sample (*n* = 982) at first no exclusion criteria were used. To be able to compare the community sample and the ED sample respondents younger than 18 years (*n* = 17) and men (*n* = 256) were excluded. Also to avoid incorporating information of people with potential ED, we excluded people with an EDE-Q score more than two standard deviations above the mean (*n* = 40) as well as one participant with a BMI of 13.9 from the community sample. This resulted in a sample of 668 women between 18 and 76 years old. *M*_age_ women was 36.03 (SD 14.17, range 18–76), self-reported *M*_BMI_ 23.72 (SD 3.80, range 16.48–43.55).

### Translation and pilot testing

The procedure of translation, back translation and cross validation of the REI was inspired by Beaton et al. (2000). The initial translation and back translation were performed independently by three students in Human Movement Sciences and a native speaker consultant. An expert committee was formed consisting of three therapists, trained and experienced in offering exercise interventions to individuals with EDs. Comparison of the translations yielded minor differences that were resolved by discussion. For the final adaptation of the Dutch version of the REI a pilot testing was conducted. Fifteen independent professionals specialized in EDs, 15 individuals with no medical or clinical background and 15 ED patients evaluated the questionnaire and gave feedback on any ambiguities. Based on this consultation, the question ‘to be attractive to the opposite sex’ (REI 19) was adjusted to: ‘to be attractive’ as it is incorrect to assume that attraction is limited to the opposite sex. The pilot testing also revealed small grammatical issues that were resolved.

### Measures

The Reasons for Exercise Inventory (Silberstein et al., 1988) is a 24 items self-report questionnaire designed to evaluate individuals reasons for exercising. Respondents rate the importance of each reason on a 7-point Likert scale ranging from 1 (*not at all important*) to 7 (*extremely important*). The REI does not use a total score, but focusses on the distribution of different reasons for exercise clustered in different factors/subscales. In the original version Cronbach’s alpha for the seven factors ranged from 0.67 to 0.81.

The Eating Disorder Examination Questionnaire (EDE-Q Fairburn and Beglin, 1994; Dutch version; Aardoom et al., 2012) is a validated and reliable self-report questionnaire to determine the severity of the ED pathology. The EDE-Q consists of 36 items of which 22 determine the total score. These 22 items comprise 4 subscales: dietary restraint, eating concern, weight concern and shape concern reported over the previous 28 days. The items are rated on a 7-point Likert scale ranging from 0 = ‘not one day’ to 6 = ‘every day’, with higher scores reflecting greater severity of ED pathology. Internal consistency of the Dutch version is good, with Cronbach’s alpha 0.95 for the total scale and varying from 0.81 to 0.91 for the subscales. In the current study Cronbach’s alpha of the total score is 0.89 in the ED sample and 0.91 in the community sample.

The Body Cathexis Scale (BCS; Secord and Jourard, 1953; Dutch version; Dorhout et al., 2015) measures the degree of satisfaction with appearance and functionality of different parts of the body. The BCS consists of 40 items rated on a 5-point Likert scale ranging from 1 = ‘very dissatisfied’ to 5 = ‘very satisfied’. Higher scores indicate higher body satisfaction. Research on the Dutch version of the BCS (Rekkers et al., 2021) in both an ED sample (*n* = 238) and a community sample (*n* = 1060) revealed three subscales: functional body satisfaction, weight related body satisfaction and non-weight related body satisfaction. Internal consistency of the Dutch version is good, Cronbach’s α = 0.90 for the total scale and Cronbach’s α = 0.83–0.85 for the subscales in a clinical sample and even higher in a community sample (Rekkers et al., 2021). In the current study Cronbach’s alpha is 0.93 in both samples.

### Statistical analyses

SPSS statistics version 28, IBM for Windows was used for exploratory factor analysis (EFA), correlations and comparisons between the two samples. The data were assessed for normality and screened for outliers. The factor structure of the REI was examined using EFA on a randomized split half of the community sample (*n* = 331) and on the ED sample. Maximum likelihood with oblique rotation was used as the factor extraction method (Costello and Osborne, 2005). Cross loadings were defined at >0.32 (Tabachnick and Fidell, 2014). A confirmatory factor analysis (CFA) using Mplus 8.0 (Muthén and Muthén, 1998–2017) was conducted on the other half of the community sample (*n* = 337) to evaluate the adequacy of the proposed factor structure following from EFA. Reported indices include root mean square error of approximation (RMSEA), with values <0.08 suggesting adequate and <0.05 good model fit (Browne and Cudeck, 1993); standardized root mean square residual (SRMR), with values between 0.05 and 0.10 indicating acceptable fit and values <0.05 indicating good fit (Schermelleh-Engel, 2003); comparative fit index (CFI) and Tucker Lewis index (TLI), with values in the range between 0.90 and 0.95 indicating good model fit (Brown, 2015).

We calculated both Cronbach’s alpha and McDonald’s omega for the subscales of the REI to ensure a comprehensive assessment of internal consistency. The levels of reliability using McDonald’s omega need to meet the standards of Cronbach’s alpha as there is no universally accepted guideline for McDonald’s omega scores (Watkins, 2017). Thus, an omega of > −0.70 is regarded as good (Cronbach, 1951; Dunn et al., 2014). To establish test-retest reliability, intra-class correlation (ICC) using two way mixed model, absolute-agreement, single measurement was calculated on data from 161 women in the community sample who completed the survey for a second time within a 3 week interval (ICC ⩾0.75 was considered excellent, between 0.60 and 0.74 good, between 0.40 and 0.59 fair and below 0.40 poor (Cicchetti, 1994).

Correlations between the REI subscales, the EDE-Q total and sub scores and the BCS were analyzed using Pearson’s product-moment correlation coefficient with correlations considered strong if *r* = 0.50–1.0, moderate if *r* = 0.30–0.49 and weak if *r* = <0.29 (Cohen, 1988). Independent *t*-tests were used to analyze differences in scores between women in the community sample and women in the ED sample. Cohen’s *d* was used to establish effect sizes.

## Results

### Exploratory factor analysis

The EFA was done independently on the ED sample and the community sample. The Kaiser-Meyer-Olkin (KMO) scale verified the sampling adequacy for the ED as well as for the community sample, with KMO = 0.820 and KMO = 0.864, respectively (Field, 2009); Barlett’s test of sphericity was statistically significant for the ED sample (χ² = 2423.534; *df* = 276, *p* < 0.000) and for the community sample (χ² = 1693.202; *df* = 207, *p* < 0.000) indicating data were suitable for EFA. Based on visual inspection of the scree plots of both samples (Watkins, 2018) and parallel analyses (Hayton et al., 2004) three factors were considered appropriate. The first factor, comprising 12 items (REI 02, 04, 06, 09, 11, 13, 16, 17, 18, 20, 22, 24), accounted for 31,22% of the total variance and was called ‘Health reasons’; the second factor, comprising 9 items (REI 01, 05, 07, 08, 12, 14, 15), explained 13,30% of the variance and was called ‘Appearance and Weight reasons’; and the third factor, formed by three items (REI 03, 10, 23) explaining 8,02% of the variance was called ‘Mood reasons’ (see Table 1).

**Table 1.** Exploratory factor analyses; item loadings on the three factors in the ED sample and the split half community sample one.

I exercise to. . .	Sample	ED (*n* = 166)			Community (*n* = 331)		
	Factor	1	2	3	1	2	3
REI 11	Improve my over-all health	**0.773**	−0.093	0.124	**0.718**	−0.094	0.029
REI 16	Improve my endurance, stamina	**0.753**	0.093	−0.026	**0.783**	−0.050	0.095
REI 17	Increase my energy level	**0.730**	0.033	−0.129	**0.621**	−0.033	−0.217
REI 24	Maintain my physical well-being	**0.718**	0.086	0.036	**0.657**	0.062	−0.142
REI 20	Have fun	**0.674**	−0.200	−0.125	**0.435**	0.147	−0.164
REI 18	Increase my resistance to illness and disease	**0.673**	−0.094	−0.135	**0.569**	0.079	−0.135
REI 09	Improve my strength	**0.647**	0.248	0.073	**0.651**	−0.197	0.146
REI 04	Improve my cardiovascular fitness	**0.633**	−0.086	−0.073	**0.457**	0.021	−0.043
REI 22	Improve my flexibility, coordination	**0.605**	0.015	−0.186	**0.583**	−0.018	−0.062
REI 02	Improve muscle tone	**0.562**	0.337	0.091	**0.657**	−0.161	0.225
REI 06	Meet new people	**0.501**	−0.147	−0.044	0.217	0.022	−0.090
REI 13	Socialize with friends	**0.464**	−0.048	0.014	0.184	0.020	−0.048
REI 05	Improve my appearance	−0.092	**0.893**	−0.062	−0.053	−**0.832**	−0.056
REI 01	Be slim	−0.279	**0.759**	−0.048	−0.042	−**0.812**	0.012
REI 14	Improve my over-all body shape	0.182	**0.731**	−0.024	0.200	−**0.692**	0.019
REI 07	Redistribute my weight	−0.040	**0.716**	−0.072	0.057	−**0.672**	−0.008
REI 21	Alter a specific area of my body	0.062	**0.683**	−0.083	0.313	−**0.377**	0.145
REI 08	Lose weight	−0.280	**0.650**	0.064	−0.163	−**0.798**	0.024
REI 19	To be attractive to others	0.268	**0.489**	−0.203	−0.006	−**0.679**	−0.138
REI 12	Be sexually desirable	0.282	**0.373**	0.111	−0.022	−**0.662**	−0.121
REI 15	To maintain my current weight	−0.149	**0.254**	−0.223	0.078	−**0.620**	−0.069
REI 03	Cope with sadness, depression	0.078	0.050	−**0.852**	−0.003	−0.204	−**0.771**
REI 23	Improve my mood	0.232	−0.056	−**0.850**	0.146	−0.092	−**0.810**
REI 10	Cope with stress, anxiety	0.131	0.055	−**0.790**	0.183	−0.100	−**0.711**

1: Health; 2: appearance/weight; 3: mood.

Boldface indicates highest factor loadings.

### Confirmatory factor analysis

In the CFA on the split half community sample two correlated errors were permitted for item 9 ‘improve my strength’ and item 2 ‘improve muscle tone’; item 13 ‘socialize with friends’ and item 6 ‘meet new people’; and item 19 ‘to be attractive to others’ and item 12 ‘to be sexually desirable’. The model with the three factors as found in the EFA and correlated errors provided an acceptable fit (see Table 2).

**Table 2.** Confirmatory factor analysis of the split half community sample two (*n* = 337).

Model	χ²	*df*	RMSEA (90% CI)	SRMR	CFI	TLI
Three factors	1362.933	267	0.115 (0.109–0.121)	0.084	0.762	0.736
Three factors with correlated errors	879.636	246	0.087(0.081–0.094)	0.073	0.865	0.848

χ^2^: chi square; *df*: degrees of freedom; RMSEA: root mean square error of approximation; 90% CI: 90% confidence interval of the RMSEA; SRMR: standardized root mean square residual; CFI: comparative fit index; TLI: Tucker Lewis index.

### Reliability

Internal consistency was excellent with Cronbach’s alpha and McDonald’s omega in the clinical ED sample: α = 0.809 and ω = 0.895 for factor 1 (REI health), α = 0.896 and ω = 0.824 for factor 2 (REI appearance/weight) α = 0.874 and ω = 0.889 for factor 3 (REI mood) and in the community sample: α = 0.901 and ω = 0.895 for factor 1 (REI health), α = 0.919 and ω = 0.824 for factor 2 (REI appearance/weight) and α = 0.907 and ω = 0.889 for factor 3 (REI mood). The ICC for factor 1 (health) was 0.80, for factor 2 (appearance/weight) 0.86 and for factor 3 (mood) 0.85.

### Group comparisons

Comparisons of the differences in scores showed that women in the community sample were significantly more likely to exercise for health reasons than those in the ED sample. Women in the ED sample were significantly more likely to exercise for appearance and weight reasons as well as for mood reasons (see Table 3).

**Table 3.** Means (*M*) and standard deviations (SD) of scores on the REI factors in the ED sample and the community sample, test of the difference and effect size (Cohen’s *d*).

REI	Community sample (*n* = 668)	ED sample (*n* = 166)	*t* (*df* = 833)	Cohen’s *d*
	*M* (SD)	*M* (SD)		
Health	4.82 (0.88)	4.09 (1.11)	−7.91**	1.20
Appearance/weight	4.12 (1.22)	5.22 (1.10)	10.58**	0.93
Mood	4.32 (1.50)	5.15 (1.42)	6.43**	1.48

REI: reasons for exercise inventory.

** *p* < 0.001.

### Correlations

Table 4 presents Pearson’s correlations of the REI factors and related measurements. In both samples we found medium positive correlations between the appearance/weight reasons for exercise and the total score of the EDE-Q and all its subscale scores. In the ED sample the scores on the subscale appearance/weight are negatively correlated with body satisfaction and those on the factor Health are positively correlated with body satisfaction. Furthermore, in the community sample very low correlations were found between body satisfaction and reasons for exercise.

**Table 4.** Pearson correlations between the REI factors and the EDE-Q and the BCS in the ED sample (*n* = 166) and the Community sample (*n* = 668).

Measures	REI health		REI appearance/weight		REI mood	
	ED	Community	ED	Community	ED	Community
EDE-Q total	−0.340**	−0.007	0.426**	0.483**	−0.002	0.094
EDE-Q R	−0.271**	0.060	0.306**	0.443**	0.012	0.048
EDE-Q EC	−0.273**	−0.031	0.399**	0.303**	−0.028	0.101
EDE-Q WC	−0.338**	−0.024	0.370**	0.435**	0.024	0.090
EDE-Q SC	−0.325**	−0.031	0.452**	0.429**	−0.022	0.089
BCS	0.319**	0.118**	−0.298**	−0.093	−0.025	−0.017

REI: reasons for exercise inventory; EDE-Q: Eating Disorder Examination Questionnaire; EDE-Q R: EDE-Q Restrictive; EDE-Q EC: EDE-Q Eating Concerns; EDE-Q WC: EDE-Q Weight Concerns; EDE-Q SC: EDE-Q Shape Concern; BCS: Body Cathexis Scale.

** p < 0.01.

## Discussion

The aim of this study was to evaluate the psychometric properties of the Dutch version of the REI in a sample of women with EDs in treatment in a specialized ED centre and a community sample of women. Differences in the reasons for exercise in women with and women without an ED were also investigated. Furthermore, associations between exercise reasons, ED symptoms and body dissatisfaction were explored.

EFA identified a three-factor model as the best fit in both samples, which was confirmed by CFA. The first factor comprises exercise for health reasons, the second exercise for appearance/weight reasons and the third exercise for mood reasons. All three factors showed good internal consistency. The 3-factor model for the 24-item Dutch version of the REI largely corresponds with the model found in studies using other non-clinical samples such as women who exercised regularly at a fitness centre or college women (Lustyk et al., 2004; O’Hara et al., 2014; Prichard and Tiggemann, 2005, 2008; Strelan et al., 2003) with the exception of the factor ‘Mood reasons’ that encompasses not five but three items directed at improving one’s mental state. REI 6 ‘to meet new people’ and REI 13 ‘to socialize with friends’, which from a face validity perspective, appear to relate to social components, loaded highest on the health factor in contrast with the English versions where these items are part of the affect/fun factor. Nevertheless, the content of these items can also be considered conceptually compatible with a broader interpretation of health-related motives, as socialization can be seen as integral component of healthy exercise motivation. As Eime et al. (2013) noted, participation in sport is often associated with enhanced psychosocial health and improved social functioning. The three factors may be used as subscales given their good internal consistency. It must be emphasized that the REI does not use a total score; the different reasons for exercise are of main interest. Therefore, the factor determination is essential in the REI.

According to Alcaraz-Ibáñez et al. (2022) reliability scores of self-report instruments are particularly sensitive to the characteristics of the study population. However, in both samples Cronbach’s alpha and McDonald’s omega showed excellent reliability scores on all three subscales. The good temporal reliability underscores the consistency of the REI, ensuring the stability of the findings over time.

The significant differences in scores between women with EDs and women in the community sample with regard to their reasons for exercise confirm earlier findings that women with EDs are more likely to exercise for appearance/weight reasons (Adkins and Keel, 2005; Brehm and Steffen, 2013) and for mood reasons (Meyer et al., 2011) and less for Health reasons. According to Halder and Mondal (2020) weight management ranks high in the motivation for exercise of the younger generation of women. Women who exercise in pursuit of an ideal body, to modify their weight or to change the shape of their body often get disappointed and discouraged (Lox et al., 2017). Individuals may set unrealistic goals and failure to meet these goals may exacerbate feelings of inadequacy and dissatisfaction (Quesnel et al., 2023). When the reason for exercise is centred around appearance and weight individuals tend to show higher levels of disordered eating and body dissatisfaction (Thome and Espelage, 2007).

The significant difference in scores on the subscale mood between women with and without ED was expected as literature states that mood regulation is an important factor for exercise reasons in people with EDs, who often experience high levels of anxiety and depression (Cosh et al., 2023). Exercise in EDs is, amongst others, used as an affect regulation strategy to improve mood and to manage negative affect (Meyer et al., 2011). Fairburn et al. (2003) mentions that the inability to appropriately cope with adverse mood states often results in the maintaining of the exercise behaviour as an affect regulation strategy. Exercise to avoid negative affect has indeed been shown to be a contributing reason for exercise in EDs (Bratland-Sanda et al., 2010; Meyer et al., 2011). It would be interesting to investigate if exercise is also used to avoid positive affect in EDs. Coniglio et al. (2019) conclude that the role of positive affect in the onset and maintenance of EDs is comparatively far less prioritized and incorporated into treatment development efforts. It is a limitation of the REI that this differentiation within mood reasons cannot be measured.

Exercising for health reasons, when executed in a balanced way, fits a healthy active lifestyle and results in mental and physical health benefits (Callaghan, 2004; Posadzki et al., 2020; Strelan et al., 2003). Women with EDs, who exercise less for health reasons, are not only dissatisfied with their appearance, they also have a lower appreciation of body functionality compared to women in community samples (Rekkers et al., 2021, 2025). Body functionality is a component of body image and defined as everything that the body does or is capable of doing (Alleva et al., 2015; Rekkers et al., 2025) which encompasses, amongst others, functions related to physical capacities and health. Promoting exercise for health reasons in clinical interventions could not only create healthier exercise behaviour, but may also stimulate a higher functionality appreciation, which can result in a more positive body image.

In both samples we found medium positive correlations between the subscale appearance/weight and both the total and subscale scores of the EDE-Q. This is in line with the hypothesis that when the disordered eating features are more prominent, people are more likely to exercise for appearance/weight reasons.

We found a negative correlation between body satisfaction and the subscale appearance/weight, indicating that when people are less satisfied with their body, they are more likely to exercise for the reason appearance/weight, possibly to improve their body shape. The subscale exercising for health reasons is positively correlated with body satisfaction in our study, which corresponds with previous research (Prichard and Tiggemann, 2008; Tiggemann and Williamson, 2000). One could argue that the potential positive influence of exercise for health reasons on the relation with one’s body emphasizes the importance of helping ED patients to shift their focus with regard to reasons for exercise. Therefore, in ED treatment there is a need to consider how to promote exercising for reasons such as health and enjoyment, as these reasons are found to be associated with positive body image outcomes. In addition, the context of the exercise is of importance as research indicates exercise in, for example, fitness centres may provoke exercise for weight and shape whereas yoga classes are potentially more focussed on exercise for health reasons (Prichard and Tiggemann, 2005).

One of the strengths of this study is the presence of a community sample as well as a clinical sample of ED patients. By studying and comparing both samples we were able to present more robust, reliable and applicable results with clear insight in earlier hypothesized differences. The similarity of our findings psychometrically underscores the robustness and cross-cultural validity of the instrument. A limitation in this study is that, to ensure comparable groups, we excluded men from the sample, so all analyses were conducted on women. Consequently, we were unable to compare differences between men and women, which is noteworthy as several studies indicate that men in general have different motivations to exercise (Craft et al., 2014; Nimiya et al., 2023). Findings in this study were collected across different DSM-5-TR diagnoses including AN, BN, BED and OSFED, but because of the small subgroups, no differentiation between women with different ED diagnoses could be made. Further research could address possible differences in reasons for exercise across diagnoses as unhealthy exercise problems are linked to a more negative prognosis of the ED (Monell et al., 2018).

## Conclusion

The Dutch version of the REI with three subscales shows good psychometric properties and supports its use for clinical and research purposes. We recommend the use of the REI in assessment and treatment of EDs, in order to be able to signal problematic exercise and to gain insight into the motives that underlie this behaviour. In this study we find that women with EDs are less likely to exercise for Health reasons compared to women in a community sample, giving rise to a new perspective on the reasons for exercise in people suffering from EDs as exercise for Health reasons contributes to a healthy active lifestyle and a positive body image.
